# Cardiac Arrhythmias and Antiarrhythmic Drugs: An Autophagic Perspective

**DOI:** 10.3389/fphys.2018.00127

**Published:** 2018-02-23

**Authors:** Joanne J. A. van Bavel, Marc A. Vos, Marcel A. G. van der Heyden

**Affiliations:** Department of Medical Physiology, Division of Heart and Lungs, University Medical Center Utrecht, Utrecht, Netherlands

**Keywords:** autophagy, AMPK, antiarrhythmic drugs, arrhythmias, mTOR, heart

## Abstract

Degradation of cellular material by lysosomes is known as autophagy, and its main function is to maintain cellular homeostasis for growth, proliferation and survival of the cell. In recent years, research has focused on the characterization of autophagy pathways. Targeting of autophagy mediators has been described predominantly in cancer treatment, but also in neurological and cardiovascular diseases. Although the number of studies is still limited, there are indications that activity of autophagy pathways increases under arrhythmic conditions. Moreover, an increasing number of antiarrhythmic and non-cardiac drugs are found to affect autophagy pathways. We, therefore, suggest that future work should recognize the largely unaddressed effects of antiarrhythmic agents and other classes of drugs on autophagy pathway activation and inhibition.

## Introduction

Degradation of cellular material occurs mainly via two pathways: the ubiquitin-proteasome system (UPS) and the autophagy-lysosome pathway. The UPS targets mainly short-lived or misfolded proteins, whereas autophagy includes the degradation, digestion and recycling of autophagy substrates, by lysosomes (Li et al., 2012). In healthy conditions, autophagy acts principally as a protective and control mechanism by maintaining cellular growth, proliferation, survival, and clearance of dying cells (Cremonese Filipi-Chiela et al., 2016). Over the last decade, an increasing amount of research has focused on autophagy and attention has been paid to the association between autophagy and cardiac diseases, including ischemia and hypertrophy. Thus far, a potential link between arrhythmic conditions and changes in autophagy activity has gained little attention, although the evidence for such interaction currently expands. There remains a need for increased research focus on the association between pro- and antiarrhythmic drugs and autophagy pathways in the heart. We review the link between cardiac autophagy and arrhythmic conditions, and the limitations regarding the effect of antiarrhythmic drugs on autophagy. Only a few years from now we can determine whether the current niche of arrhythmias and autophagy research will emerge in a mature field of investigation.

## Autophagy types and targets

**Figure 1:**
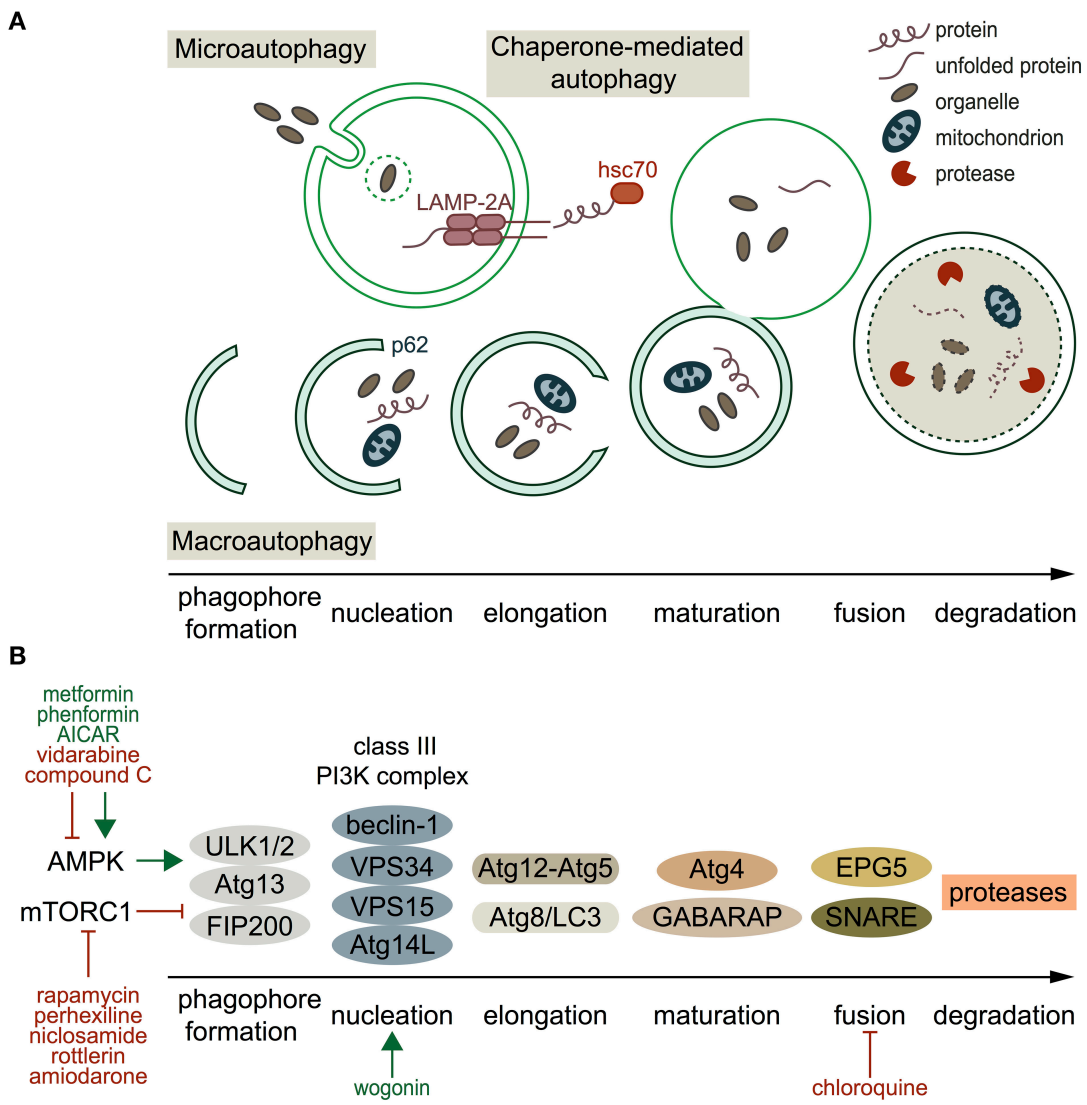
Autophagy types, processes, molecular players and pharmacological regulators. **(A)** Schematic presentation of the three autophagy types. Microautophagy refers to the direct uptake of soluble cellular substrates from the cytoplasm by invaginations in lysosomal membranes. Chaperone-mediated autophagy includes targeting of a specific motif in a substrate protein, translocation of the substrate to the lysosome by heat shock-cognate protein of 70 kDa (hsc70), and translocation into the lysosome by lysosome-associated membrane protein 2A (LAMP-2A) on the lysosomal membrane. Macroautophagy starts with formation of a double-membraned phagophore, at which proteins and lipids are recruited by, among others, p62 (nucleation). Then, the phagophore elongates, matures, and the autophagosome fuses with a lysosome. **(B)** Overview of the mediators involved in macroautophagy and some of their stimulators/inhibitors (green and red respectively). Autophagy initiation occurs by activated AMPK, which phosphorylates the ULK1/2-Atg13-FIP200 complex. mTORC1 inhibits this complex. Once the autophagy pathway is activated, a phagophore is formed and the class III PI3K complex is responsible for nucleation. Two different conjugation systems are important for elongation of the phagophore (Atg12-Atg5 and Atg8/LC3). GABARAP and Atg4 are involved in autophagosome maturation, and fusion of the autophagosome with a lysosome is mediated by EPG5 and SNARE proteins. Finally, the cargo is degraded by proteases.

Autophagy substrates (referred to as cellular material) include proteins, proteasomes, lysosomes, endoplasmic reticulum (ER), mitochondria, lipid droplets, polyribosomes, peroxisomes, bacteria, viruses and ruptured phagosomes. These substrates are targeted for degradation as long as they are freely accessible in the cytosol (Galluzzi et al., 2017). Three types of autophagic targeting are recognized (Figure 1A). Microautophagy is the least studied type of autophagy, which involves the direct uptake of soluble cellular substrates from the cytoplasm by invaginations in lysosomal membranes (Li et al., 2012). Macroautophagy, the best characterized variant of autophagy, is used synonymous with the term autophagy. It refers to both selective and non-selective capture of cellular components in double-membraned vesicles in the cytosol, and subsequent transport of the content to lysosomes (Feng et al., 2014). Chaperone-mediated autophagy (CMA) is a selective autophagic mechanism, which is mediated by the recognition of a peptide sequence in substrate proteins by chaperones, e.g. hsc70 (Cuervo and Wong, 2014). The substrate binds to lysosome-associated membrane protein 2A (LAMP-2A) and translocates into the lysosome for degradation. The main and most important function of the strictly organized process of autophagy is to maintain cellular homeostasis (Singh and Cuervo, 2011). Autophagy can act selectively, which includes the degradation of specific cargo selected by receptor proteins, and non-selectively by maintenance of intracellular nutrient supply with starvation as its main trigger (Svenning and Johansen, 2013). Besides small cellular components, selective autophagy can target specific cell components, e.g. mitochondria (mitophagy), ER (reticulophagy), ribosomes (ribophagy), lipids (lipophagy), and ion channels (channelophagy, as suggested by Klionsky et al., 2007; Kondratskyi et al., 2017). Autophagic processes are either continuously active (constitutive) or triggered (inducible). The primary stimulus of autophagy in yeast is nutrient withdrawal, whereas in mammals several stimuli can trigger autophagy, of which the most important are nutritional changes (e.g. starvation), organelle damage, hormonal regulation, infectious agents, hypoxia (such as in cardiac ischemia), and intracellular accumulation of toxic products (e.g. some antiarrhythmic drugs; Kudenchuk et al., 1984; Singh and Cuervo, 2011; Reggiori and Klionsky, 2013). The first reports on autophagic processes appeared in 1955. Christian de Duve coined the term “autophagy” in 1963, which was based on the discovery of self-eating lysosomes in rat hepatic cells (De Duve et al., 1955). The following decades, research focused on studying molecular autophagy mechanisms in yeast, which differ from autophagy processes in mammals (Matsuura et al., 1997; Reggiori and Klionsky, 2013). Furthermore, genetic screens revealed the existence of AuTophaGy-related (ATG) genes, of which the role in autophagy is associated to various processes in health and disease (Feng et al., 2014; Schneider and Cuervo, 2014). Recently, the field of autophagy was honored by a Nobel Prize awarded to Yoshinori Ohsumi in 2016 (Levine and Klionsky, 2017).

## Autophagy-related proteins and their use as therapeutical targets

ATG proteins are mainly described in macroautophagy because of its best-known machinery, and are illustrated in Figure 1B. Two important regulators of autophagy initiation are AMP-activated protein kinase (AMPK) and mechanistic target of rapamycin complex 1 (mTORC1). Active AMPK stimulates autophagy initiation, whereas mTORC1 inhibits autophagy (Kim et al., 2011). In autophagy stimulation, the ULK1/2-Atg13-FIP200 complex becomes active, ATG proteins are recruited and a phagophore is formed (Xie et al., 2015). The class III phosphatidylinositol 3-kinase (PI3KCIII) complex (beclin-1, VPS34, VPS15, and Atg14L) mediates nucleation of the phagophore (Baskaran et al., 2014). Different ubiquitin-like conjugation systems (Atg12-Atg5 and Atg8/LC3) are central in the next autophagic step; elongation of the phagophore, and proteins and lipids involved in autophagosome formation and maturation are recruited by the PI3KCIII complex (Feng et al., 2014; Xie et al., 2015). Beclin-1 is part of the PI3KCIII complex, and plays a key role in autophagosome formation and maturation (Xie et al., 2015). Mature autophagosomes move along microtubules and fuse with lysosomes in a process that involves gamma-aminobutyric acid receptor-associated proteins (GABARAP) and soluble NSF attachment protein receptor (SNARE) family proteins (Nair et al., 2011; Itakura et al., 2012). Autophagy's final step is degradation of the autophagolysosome cargo by different types of proteases (Kaminskyy and Zhivotovsky, 2012). Detailed overviews of the macroautophagy machinery are given by Feng et al. (2014) and Xie et al. (2015).

Inability to maintain cellular homeostasis due to defective autophagy associates with a variety of systemic diseases, such as cancer, neurodegeneration, liver disease and (cardio-) myopathies (Schneider and Cuervo, 2014). Distinct ATG proteins are considered as interesting targets for autophagy modulation (Figure 1B, Table 1), in which the field of cancer therapy progressed furthest (Mulcahy Levy et al., 2017). Rapamycin and its analogs (rapalogs) inhibit mTORC1 resulting in autophagy activation, and are used in certain cancer treatments (Li J. et al., 2014). Other recognized mTORC1 inhibitors are perhexiline (antianginal agent), niclosamide (used in treating worm infections), and rottlerin (natural product, known to open potassium channels) (Balgi et al., 2009). Activation of autophagy can also result from AMPK activators, such as commonly used antidiabetic agents metformin and phenformin, and AMP analog 5-aminoimidazole-4-carboxamide-1-β-D-ribofuranoside (AICAR) (Zhou et al., 2001; Yang et al., 2013; Ducommon et al., 2014). Common inhibitors of AMPK include adenine 9-beta-D-arabinofuranoside (Ara-A, or vidarabine) and compound C (Zhou et al., 2001; Pelletier et al., 2005). Antitumor drug wogonin can affect the next step in the autophagy pathway (phagophore nucleation) by targeting the beclin-1/PI3K complex, and thereby inducing autophagy in human pancreatic cancer cells (Li et al., 2016). The antimalarial and anti-inflammatory agent chloroquine targets the last step in the autophagy pathway by inhibiting fusion of autophagosomes and lysosomes (Yoon et al., 2010). Although certain compounds seem to target autophagy pathways effectively, former mentioned compounds can act directly with a positive effect (e.g., tumor regression), as well as indirectly causing side effects.

**Table 1:** The effect of the discussed compounds on autophagy activation, their effective concentration on autophagy regulation, their therapeutic plasma concentrations, and their potential link to arrhythmic conditions.

**Compound**	**Autophagic activation**	**Concentrations**		**Associated with arrhythmias^a^**	**References**
AICAR	↑	AMPK activation Plasma levels	500 μM not reported	+ hERG inhibition	Zhou et al., 2001; Almilaji et al., 2013
Amiodarone	↑	mTORC1 inhibition Plasma levels	>10 μM 0.8–3.9 μM	+ QT prolongation	Balgi et al., 2009; Tarapués et al., 2014; Hrudikova Vyskocilova et al., 2017.
Chloroquine	↓	Autolysosome fusion ↓ Mean peak plasma level	120 μM 0.4 μM	+ at high dose	Walker et al., 1983; White, 2007; Yoon et al., 2010
Compound C	↓	AMPK inhibition Plasma levels	20 μM not reported	↓(potentially) by hERG activation	Zhou et al., 2001; Almilaji et al., 2013
Dronedarone	↑	Autophagy activation Steady-state plasma levels	2 μM 0.15–0.3 μM	+ QT prolongation	Wadhani et al., 2006; Patel et al., 2009; Piccoli et al., 2011
Metformin	↑	AMPK activation Steady-state plasma levels	>2 μM 10–40 μM	↓AF in DM patients	Zhou et al., 2001; Chang et al., 2014
Niclosamide	↑	mTORC1 inhibition Serum concentration range	1 μM 0.76–18.32 μM	Not reported	Andrews et al., 1982; Balgi et al., 2009
Nifedipine	↑	Autophagy activation Mean peak plasma level	10 μM 0.35 μM	↓ LTCC blocker	Van Bortel et al., 1989; Redfern et al., 2003; Pushparaj et al., 2015
Paliperidone	↑	Downstream effector mTOR Mean plasma level	not reported 84.4 nM	+ hERG inhibition	Nazirizadeh et al., 2010; Vigneault et al., 2011; Mas et al., 2015
Pentamidine	↑	Kir2.1 degradation Plasma levels	5–10 μM 0.5–2.4 μM	+ Risk for TdP	Waalkes et al., 1970; Antoniou and Gough, 2005; Nalos et al., 2011
Perhexiline	↑	mTORC1 inhibition Serum concentration range	1–10 μM 0.8–3.8 μM	+ hERG inhibition	Plicher et al., 1985; Walker et al., 1999; Balgi et al., 2009
Phenformin	↑	AMPK activation Plasma level	>0.3 mM 0.27 μM	+ hERG inhibition	Marchetti et al., 1987; Almilaji et al., 2013; Vincent et al., 2015
PI3K inhibitors^b^	↑	AMPK activation^c^ Mean peak plasma level^c^	5 μM 3.6 μM	+ APD prolongation	Fava et al., 2008; Yu et al., 2013; Cohen et al., 2017
Propranolol	↓	Late block in autophagy Plasma levels	10 μM 20–428 μM	↓shorter QT in LQT1	Castleden et al., 1975; Farah et al., 2014; Ahn et al., 2017
Ranolazine	↑	Autophagy activation Mean steady-state level	1 μM 6 μM	↓ AF episodes	CV Therapeutics Inc., 2007; Huang et al., 2010; Guerra et al., 2017
Rapamycin	↑	mTORC1 inhibition Target concentration range	0.5–100 nM 4–22 nM	+ >28 nM	Stenton et al., 2005; Foster and Toschi, 2009; Karakas et al., 2016
Rottlerin	↑	mTORC1 inhibition Plasma levels	1–3 μM not reported	+ APD shortening	Lu et al., 2008; Balgi et al., 2009
Verapamil	↑	Autophagy activation Mean peak plasma level	1 μM 0.8 μM	+ LTCC and hERG inhibition	Frishman et al., 1982; Redfern et al., 2003; Williams et al., 2008
Vidarabine	↓	AMPK inhibition Mean peak plasma level	>0.5 mM 3.7 μM	–	Whitley et al., 1980; Pelletier et al., 2005; Wada et al., 2016
Wogonin	↑	Beclin-1/PI3K activation Plasma levels	40 μM not reported	↓ in ischemic model	Lee et al., 2011; Li et al., 2016

AF, atrial fibrillation; APD, action potential duration; DM, diabetes mellitus; LQT1, long QT syndrome 1; LTCC, L-type calcium channel; TdP, Torsade de Pointes; Compound association with arrhythmias (+), no association (–), preventive effect (↓) or “not reported.”

a *Arrhythmic associations tested in human, animal, or in vitro models*.

b *PI3K inhibitor examples are, as mentioned in the text, nilotinib, dasatinib, and sunitinib*.

c *Nilotinib concentration*.

## Autophagy in the heart: some lessons from gene defects

Autophagy is important for maintenance of the highly organized cardiac structure, function and homeostasis. Nutrient insufficient conditions lead to the inactivation of mTORC1: rapamycin interacts with and inhibits mTORC1 activating unc-51-like kinase 1 and 2 (ULK1 and ULK2), leading to autophagy induction (Lavandero et al., 2013). Activation of sensitive nutrient sensor AMPK occurs when ATP/AMP levels decrease due to exercise, ischemia or lack of glucose, resulting in autophagy initiation (Hardie et al., 2012). Other important autophagy mediators in the cardiovascular system include inositol 1,4,5-triphosphate (IP_3_), transcription factor 53 (TP53), cyclic AMP-dependent protein kinase A (PKA), histone acetyltransferases (HATs) and histone deacetylases (HDACs), glycogen synthase kinase 3β (GSK3β), nicotinamide adenine dinucleotide (NAD^+^) and microRNAs, which can initiate and inhibit autophagy, as reviewed by Lavandero et al. (2013). Impaired autophagy pathway signaling, due to ATG protein deficiency, may cause cardiac pathologies. Mice with Atg5 and Atg7 deficiency, ATG proteins involved in phagophore elongation, displayed dilated cardiomyopathy and contractile dysfunction, and accumulation of defective proteins and organelles, respectively (Komatsu et al., 2005; Nakai et al., 2007). Ectopic P-granules autophagy protein 5 (EPG5), important in translocation of autophagosomes to lysosomes, deficient individuals suffer from the Vici syndrome; a multisystem disorder with autophagy malfunction (Cullup et al., 2013). Cardiac symptoms, predominantly hypertrophy and left ventricular dilatation, are present in 90% of the Vici syndrome patients (Byrne et al., 2016). Deficiency of LAMP-2, required for the fusion of autophagosomes with endosomes and lysosomes (Endo et al., 2015), leads to Danon disease causing intellectual disability, skeletal myopathy and severe cardiomyopathy (D'souza et al., 2014). LAMP-2 deficient mice have a high mortality rate, show autophagosome accumulation in the pancreas, liver, kidney, skeletal muscle and heart, and possess cardiomyocytes filled with large vacuoles. The latter might be the cause of a reduced heart muscle contractility observed in these mice (Tanaka et al., 2000). Apart from the reductive, but instructive, ATG deficient models, most studies however focus on ischemia/reperfusion (I/R) and hypertrophy models to examine the role of cardiac autophagy (Lavandero et al., 2013).

## Autophagy in cardiac ischemia/reperfusion and hypertrophy models

Both protective (clearance and removal of misfolded proteins and roles in energy homeostasis) and detrimental (massive digestion of cellular components and cross-talk to other forms of cell death) functions of autophagy have been presented in ischemic and hypertrophy models (Sciarretta et al., 2011). In the ischemic heart, upregulation of autophagy associates with a reduction in infarct size and apoptosis, and vice versa, suggested to work cardioprotective by maintaining energy levels (Sciarretta et al., 2014). In this situation, the drop in ATP levels during ischemia activates AMPK leading to an upregulation of autophagy activation (Takagi et al., 2007). In mice hearts however that underwent I/R, when oxygen and nutrient supply is impaired and subsequently restored, AMPK is inactivated, mTOR is upregulated, and beclin-1 is highly upregulated, suggesting an inhibition of autophagy (Matsui et al., 2007). Remarkably, these mice showed an increase in autophagosome levels and a decrease in apoptosis.

Cardiac hypertrophy is associated with an upregulation of autophagy. Weng et al. (2014) presented an increase in autophagic gene (Atg5 and Atg16) expression, and increased beclin-1 and microtubule-associated protein 1A/1B chain 3 phosphatidylethanolamine conjugate (LC3-II) levels in transverse aortic constriction (TAC), to induce pressure overload, operated mice. Furthermore, AMPK induces autophagy in TAC operated hearts by regulating the mTORC1 pathway, leading to inhibition of cardiac hypertrophy and improved cardiac function (Li Y. et al., 2014). Cardiac-specific knockout of mTOR showed an impaired hypertrophic response and enhanced heart failure progression after TAC mediated pressure-overload in mice, suggesting detrimental effects when mTOR is lacking (Zhang et al., 2010). However, pharmacologic inhibition of mTOR indicates a cardiac protective effect: rapamycin treatment in TAC operated mice showed an inhibitory effect on cardiac hypertrophy development (McMullen et al., 2004). Moreover, treatment of rapamycin in cardiac transplant recipients resulted in reduced left ventricular mass and improved diastolic function (Raichlin et al., 2008). Altogether, current evidence states that regulation of autophagy levels is crucial in protecting cardiomyocytes under ischemic and hypertrophic conditions.

## Increased autophagy activation in arrhythmic conditions

Connexin43 (Cx43) proteins form gap junctions, which are responsible for the propagation of cardiac action potentials between cardiomyocytes. In the recent years, literature has been growing regarding the role of autophagy in degradation of Cx43 (Falk et al., 2014). A lowered expression of Cx43 was also linked to arrhythmias: among others, (Shu et al., 2017) showed a lowered Cx43 protein expression in a canine model with atrial fibrillation (AF) induced by rapid atrial pacing (RAP). It remains to be identified if a lowered Cx43 expression in arrhythmic conditions was mediated by autophagic degradation. As an indication of autophagy activation, levels of autophagy mediators have been examined in several arrhythmic conditions. A RAP canine model, vulnerable to AF, showed an increase in LC3B-II (LC3 family member) and phosphorylated AMPK (p-AMPK) protein levels (Yuan et al., 2014). Moreover, in the same study, it was presented that these autophagy mediators were increased in human patients suffering from AF. Furthermore, beclin-1 and LC3B-II expression levels were increased in I/R-injured fibrillated mouse hearts (Meyer et al., 2013). The increased levels of these autophagy mediators suggest increased autophagy activation in response to arrhythmias. Although the number of autophagic vacuoles was elevated in patients who underwent coronary artery bypass grafting with postoperative AF, decreased levels of LC3B-II levels were found in these patients (Garcia et al., 2012). A decrease in vesicle degradation and an impaired autophagic flow can clarify the elevated vesicle levels and the decreased LC3B-II levels, respectively (Garcia et al., 2012). These exciting findings, albeit just a few, appear to outpoint that autophagy pathways are becoming activated in arrhythmic conditions, and are therefore of interest as potential therapeutic targets. Nonetheless, the exact mechanistic relationship between autophagy and cardiac arrhythmias clearly remains to be elucidated.

## Antiarrhythmic drugs affect autophagy pathways

Along with limited research into autophagy occurrence and regulation in arrhythmic conditions, only a few studies have thus far focused on the effect of antiarrhythmic drugs on autophagy activation and the results are interesting. Table 1 demonstrates the effect on autophagic regulation, the effective concentrations affecting autophagy, the clinical effective concentrations, and a possible association with arrhythmias of the compounds discussed in this perspective. The effective concentrations of the compounds on autophagy regulation, although tested *in vitro* with limited concentration ranges, can be compared to drug plasma levels in patients. Overlapping concentrations indicate that a number of clinically used compounds are likely to influence authophagy regulation. Antiarrhythmic drugs are classified by the effect on the targets whose actions form the cardiac action potential. Class I drugs block Na^+^ channels, class II drugs are adrenergic receptor antagonists, class III drugs are K^+^ channel blockers, and the AV-node conduction is slowed down by class IV drugs usually by blockage of L-type calcium channels (LTCCs). The antiarrhythmic effects of Na^+^ channel blocker ranolazine, initially developed as antianginal agent, are convincing (Gupta et al., 2015). Huang et al. (2010) showed induced autophagy activation after ranolazine treatment in HL-1 cells and isolated rat cardiomyocytes. Commonly used beta-blocker propranolol is reported as autophagy inhibitor: increased LC3-II levels, autophagosome formation, and p62 (which degrades during autophagy stimulation) levels were measured in HepG2 cells, suggesting an inhibition of hepatic autophagy by propranolol at a later stage due to reduced degradation (Farah et al., 2014). Class III antiarrhythmic drug amiodarone inhibits mTORC1 leading to stimulation of the autophagy pathway, which was explored *in vitro* (Balgi et al., 2009). In another *in vitro* study, we indicated lysosomal impairment by amiodarone and its synthetic analog dronedarone, which resulted in increased inward rectifier potassium channel K_ir_2.1 expression and intracellular accumulation (Ji et al., 2017a). The well-known drawback of amiodarone is its high incidence of side effects, including thyroid toxicity, pulmonary toxicity, hepatic toxicity, neurological toxicity, which seem to be related to the lifetime cumulative dose of the drug (Santangeli et al., 2012). However, pharmacological activation of autophagy by amiodarone has been shown to improve liver regeneration after partial hepatectomy in mice (Lin et al., 2015). LTCC blocker nifedipine, used as arterial vasodilator, increases autophagic flow, as shown by increased presence of autophagosomes and LC3-II levels, and lower p62 levels in isolated rat cardiomyocytes (Pushparaj et al., 2015). LTCC blocker verapamil, used in treating angina and arrhythmias, increases autophagic flux, which was shown by elevated LC3-II levels in PC12 cells and in a series of human cell lines, in which the latter also included increased development of autophagic vacuoles (Williams et al., 2008; Kania et al., 2017). These studies, although limited in number, clearly represent the existing link between antiarrhythmic drugs and autophagy, and the direct outcomes can be both activation and inhibition of autophagy.

## Non-cardiac drugs can act proarrhythmic and affect autophagy

As well as antiarrhythmic compounds, non-cardiac drugs can have the tendency to act proarrhythmic, e.g. by prolonging the QT interval with an increased risk for Torsade de Pointes (TdP) arrhythmias (Bossu et al., 2016), and they can affect the autophagic pathway. Many compounds with increased proarrhythmic risk are clinically used, or only reached phase I of clinical trials, to treat various disease areas. The former discussed drug chloroquine, which increases the lysosomal pH and thereby prevents the degradation of certain autophagy substrates, is reported as proarrhythmic. Accumulated levels of K_ir_2.1 were found intracellularly and I_K1_ densities increased due to chloroquine treatment (Jansen et al., 2008). From an autophagic perspective these results can be linked to the chloroquine-induced QT prolongation, conduction disturbances and cardiomyopathy at high doses, as reviewed by White (2007). The proarrhythmic effect of antiprotozoal drug pentamidine has been firstly reported in 1987 by the description of two case reports with occurrence of TdP arrhythmias after administration of pentamidine, which results have been confirmed later on (Wharton et al., 1987; Antoniou and Gough, 2005). We suggested a link between pentamidine and autophagy, in which pentamidine may induce lysosomal degradation of potassium channel K_ir_2.1 (Nalos et al., 2011). Pentamidine analogs have been, and still are, tested to finally develop efficient and specific K_ir_2.x ion-channel-carried inward rectifier current (I_k1_) inhibitors for treating atrial fibrillation and short QT syndrome type 3 (Takanari et al., 2013; Ji et al., 2017b). Antipsychotic drug paliperidone, which inhibits human ether-a-go-go-related gene (hERG) K^+^ channel, has also been characterized to increase the QT interval and increase the risk for TdP arrhythmia (Vigneault et al., 2011; Hagiwara et al., 2016). It may be assumed that it also affects autophagy, since mTOR was identified as a downstream effector of paliperidone-induced extrapyramidal symptoms (side effect of antipsychotics), as observed in a network analysis of gene expression (Mas et al., 2015). The role of autophagy in cancer has been characterized as paradoxical because of its pro-survival and pro-death outcomes (Helgason et al., 2013). A frequently altered pathway in cancer includes PI3K and its inhibitors seem to treat solid tumors and hematologic malignancies (Mayer and Arteaga, 2016). Nilotinib, dasatinib and sunitinib are examples of PI3K inhibitors, which are approved by the FDA to treat certain cancer types, and are shown to induce autophagy pathways in cancer cell models (Le et al., 2010; Yu et al., 2013; Wang et al., 2017). However, a recent study by Cohen et al. (2017), presented a prolongation of the action potential by former named PI3K inhibitors, and thereby clearly suggests that drug safety testing should be improved. The compounds discussed in this paragraph, either approved by the FDA or currently in clinical trials, seem to affect the autophagy pathway and cardiac action potential, while their original purpose is not to affect those. Causality however, needs to be demonstrated.

## Conclusion and future perspectives

Autophagy regulation is crucial in basal and diseased conditions, and has been shown to act both protective and detrimental in cardiac disease models. Up to now, evidence has brought forward that autophagy activation changes in arrhythmic conditions of the heart. In addition, some antiarrhythmic drugs have been shown to affect autophagy pathways and this may associate with adverse effects. The direct effects and deciphering of the complex underlying mechanisms of antiarrhythmic drugs on autophagy mediation in the heart remain to be determined. Ion channels are crucial in maintaining a regular cardiac rhythm and some are also involved in autophagy regulation, as reviewed by Kondratskyi et al. (2017), indicating a possible direction of future research. Another research aim should be to understand the dependent role of remodeling on autophagy in cardiac arrhythmic conditions. Gene defect models and arrhythmia-induced models are promising in understanding the mechanistic relationship between autophagy and arrhythmias. We also suggest that future work should include the examination of autophagy effects in exploring the effectiveness of antiarrhythmic drugs. This may improve drug development to provide safer antiarrhythmic drugs by removal of autophagy pathway disturbances. Antiarrhythmic drugs may then not further worsen autophagy dysregulation in arrhythmic conditions. Beyond doubt, from an autophagic perspective; focus should increase on its regulation under arrhythmic conditions, and on the effects of its unknown targeting by antiarrhythmic compounds and other drugs.

## Author contributions

All authors listed, have made substantial, direct and intellectual contribution to the work, and approved it for publication.

### Conflict of interest statement

The authors declare that the research was conducted in the absence of any commercial or financial relationships that could be construed as a potential conflict of interest.

## Abbreviations

AF: atrial fibrillation
AMPK: AMP-activated protein kinase
AICAR: 5-aminoimidazole-4-carboxamide-1-β-D-ribofuranoside
Ara-A: adenine 9-beta-D-arabinofuranoside
ATG: AuTophaGy related
CMA: chaperone-mediated autophagy
Cx43: connexin43
EPG5: ectopic P-granules autophagy protein 5
ER: endoplasmic reticulum
GABARAP: gamma-aminobutyric acid receptor-associated proteins
GSK3β: glycogen synthase kinase 3β
HAT: histone acetyltransferase
HDAC: histone deacetylase
hERG: human ether-a-go-go-related gene
hsc70: heat shock-cognate protein of 70 kDa
IP_3_: inositol 1,4,5-triphosphate
I/R: ischemia/reperfusion
LAMP-2A: lysosome-associated membrane protein 2A
LC3-II: microtubule-associated protein 1A/1B chain 3 phosphatidylethanolamine conjugate
LTCC: L-type calcium channel
mTOR: mechanistic target of rapamycin
mTORC1: mTOR complex 1
NAD^+^: nicotinamide adenine dinucleotide
P-AMPK: phosphorylated AMPK
PI3KCIII: class III phosphatidylinositol 3-kinase
PKA: protein kinase A
RAP: rapid atrial pacing
SNARE: soluble NSF attachment protein receptor
TAC: transverse aortic constriction
TdP: torsade de pointes
TP53: transcription factor 53
ULK1/2: unc-51-like kinase 1 and 2
UPS: ubiquitin proteasome system.
